# 2,6-Dichloro­phenyl 4-methyl­benzoate

**DOI:** 10.1107/S1600536808009616

**Published:** 2008-04-16

**Authors:** B. Thimme Gowda, Sabine Foro, K. S. Babitha, Hartmut Fuess

**Affiliations:** a Department of Chemistry, Mangalore University, Mangalagangotri 574 199, Mangalore, India; b Institute of Materials Science, Darmstadt University of Technology, Petersenstrasse 23, D-64287 Darmstadt, Germany

## Abstract

The structure of the title compound (26DCP4MeBA), C_14_H_10_Cl_2_O_2_, resembles those of phenyl benzoate (PBA), 2,6-dichloro­phenyl benzoate (26DCPBA), 2,4-dichloro­phenyl 4-methyl­benzoate (24DCP4MeBA) and other aryl benzoates, with similar bond parameters. The dihedral angle between the benzene and benzoyl rings in 26DCP4MeBA is 77.97 (9)°, compared with values of 55.7 (PBA), 75.75 (10) (26DCPBA) and 48.13 (5)° (24DCP4MeBA). The mol­ecules in the title compound are packed into zigzag chains in the *bc* plane.

## Related literature

For related literature, see: Adams & Morsi (1976 ▶); Gowda *et al.* (2007*a* ▶,*b* ▶); Nayak & Gowda (2008 ▶).
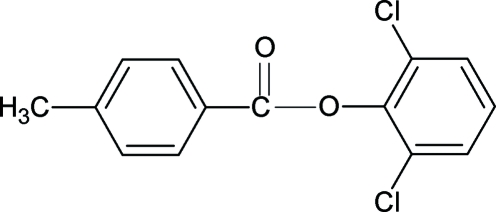


## Experimental

### 

#### Crystal data

C_14_H_10_Cl_2_O_2_
*M* *_r_* = 281.12
Monoclinic, 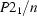

*a* = 9.5688 (8) Å
*b* = 11.1370 (9) Å
*c* = 13.1947 (9) Å
β = 108.898 (7)°
*V* = 1330.33 (18) Å^3^
*Z* = 4
Cu *K*α radiation
μ = 4.32 mm^−1^
*T* = 299 (2) K
0.60 × 0.35 × 0.30 mm

#### Data collection

Enraf–Nonius CAD-4 diffractometer
Absorption correction: ψ scan (North *et al.*, 1968 ▶) *T*_min_ = 0.202, *T*_max_ = 0.273
3035 measured reflections
2366 independent reflections
2025 reflections with *I* > 2σ(*I*)
*R*_int_ = 0.040
3 standard reflections frequency: 120 min intensity decay: none

#### Refinement

*R*[*F* ^2^ > 2σ(*F* ^2^)] = 0.049
*wR*(*F* ^2^) = 0.168
*S* = 1.01
2366 reflections
165 parameters
H-atom parameters constrained
Δρ_max_ = 0.36 e Å^−3^
Δρ_min_ = −0.43 e Å^−3^

### 

Data collection: *CAD-4-PC* (Enraf–Nonius, 1996 ▶); cell refinement: *CAD-4-PC*; data reduction: *REDU4* (Stoe & Cie, 1987 ▶); program(s) used to solve structure: *SHELXS97* (Sheldrick, 2008 ▶); program(s) used to refine structure: *SHELXL97* (Sheldrick, 2008 ▶); molecular graphics: *PLATON* (Spek, 2003 ▶); software used to prepare material for publication: *SHELXL97*.

## Supplementary Material

Crystal structure: contains datablocks I, global. DOI: 10.1107/S1600536808009616/om2223sup1.cif

Structure factors: contains datablocks I. DOI: 10.1107/S1600536808009616/om2223Isup2.hkl

Additional supplementary materials:  crystallographic information; 3D view; checkCIF report

**Fig. 3.**
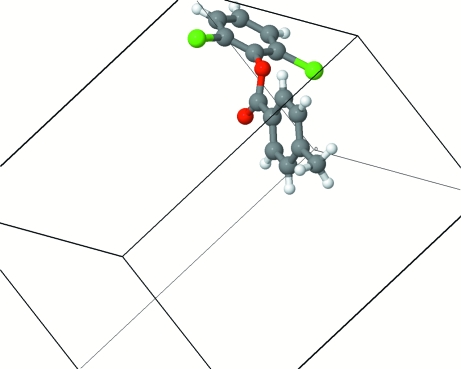
A view of the title molecule and unit cell

Enhanced figure: interactive version of Fig. 3

## Figures and Tables

**Fig. 1.**
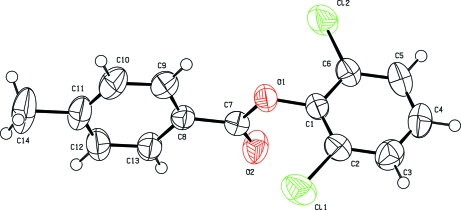
Molecular structure of the title compound, showing the atom labeling. Displacement ellipsoids are drawn at the 50% probability level.

**Fig. 2.**
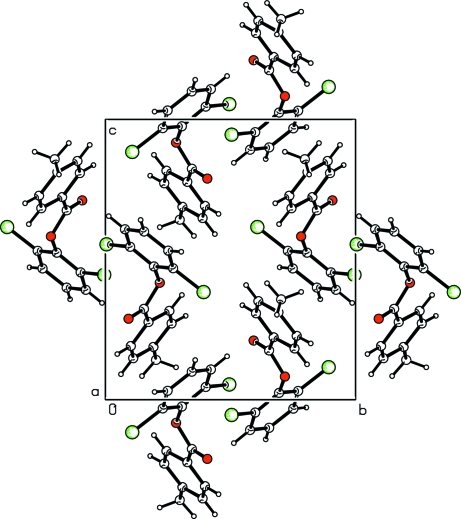
Molecular packing of the title compound as viewed down a.

## supplementary crystallographic information

### Comment

In the present work, as part of a study of the substituent effects on the structures of chemically and industrially significant compounds (Gowda *et al.*, 2007*a*,*b*), the structure of 2,6-dichlorophenyl 4-methylbenzoate (26DCP4MeBA) has been determined. The structure of 26DCP4MeBA (Fig. 1) resembles those of phenyl benzoate (PBA) (Adams & Morsi, 1976), 2,6-dichlorophenyl benzoate (26DCPBA) (Gowda *et al.*, 2007*a*), 2,4-dichlorophenyl 4-methyl benzoate (24DCP4MeBA) (Gowda *et al.*, 2007*b*) and other aryl benzoates. The bond parameters in 26DCP4MeBA are similar to those in PBA, 26DCPBA, 24DCP4MeBA and other benzoates. The dihedral angle between the benzene and benzoyl rings in 26DCP4MeBA is 77.97 (9)°, compared to the values of 55.7° (PBA)(Adams & Morsi, 1976), 75.75 (10)° (26DCPBA)(Gowda *et al.*, 2007*a*) and 48.13 (5)° (24DCP4MeBA)(Gowda *et al.*, 2007*b*). The molecules in 26DCP4MeBA are packed into a zigzag structure with the dichlorophenyl ring being nearly orthogonal to the benzoyl ring (Fig. 2).

### Experimental

The title compound was prepared according to a literature method (Nayak & Gowda, 2008). The purity of the compound was checked by determining its melting point. It was characterized by recording its infrared and NMR spectra (Nayak & Gowda, 2008). Single crystals of the title compound were obtained by slow evaporation of an ethanolic solution.

### Refinement

The H atoms were positioned with idealized geometry using a riding model (C—H = 0.93–0.96 Å) with *U*_iso_ = 1.2 *U*_eq_ of the parent atom.

### Crystal data

**Table:**

C_14_H_10_Cl_2_O_2_	*F*_000_ = 576
*M**_r_* = 281.12	*D*_x_ = 1.404 Mg m^−^^3^
Monoclinic, *P*2_1_/*n*	Cu *K*α radiation λ = 1.54180 Å
Hall symbol: -P 2yn	Cell parameters from 25 reflections
*a* = 9.5688 (8) Å	θ = 5.3–18.7º
*b* = 11.1370 (9) Å	µ = 4.32 mm^−^^1^
*c* = 13.1947 (9) Å	*T* = 299 (2) K
β = 108.898 (7)º	Rod, colourless
*V* = 1330.33 (18) Å^3^	0.60 × 0.35 × 0.30 mm
*Z* = 4	

### Data collection

**Table:**

Enraf–Nonius CAD-4 diffractometer	*R*_int_ = 0.040
Radiation source: fine-focus sealed tube	θ_max_ = 67.0º
Monochromator: graphite	θ_min_ = 5.0º
*T* = 299(2) K	*h* = −11→2
ω/2θ scans	*k* = −13→0
Absorption correction: ψ scan(North *et al.*, 1968)	*l* = −15→15
*T*_min_ = 0.202, *T*_max_ = 0.273	3 standard reflections
3035 measured reflections	every 120 min
2366 independent reflections	intensity decay: none
2025 reflections with *I* > 2σ(*I*)	

### Refinement

**Table:**

Refinement on *F*^2^	Hydrogen site location: inferred from neighbouring sites
Least-squares matrix: full	H-atom parameters constrained
*R*[*F*^2^ > 2σ(*F*^2^)] = 0.049	*w* = 1/[σ^2^(*F*_o_^2^) + (0.1211*P*)^2^ + 0.3061*P*] where *P* = (*F*_o_^2^ + 2*F*_c_^2^)/3
*wR*(*F*^2^) = 0.168	(Δ/σ)_max_ = 0.011
*S* = 1.01	Δρ_max_ = 0.36 e Å^−^^3^
2366 reflections	Δρ_min_ = −0.43 e Å^−^^3^
165 parameters	Extinction correction: SHELXL97 (Sheldrick, 2008), Fc^*^=kFc[1+0.001xFc^2^λ^3^/sin(2θ)]^-1/4^
Primary atom site location: structure-invariant direct methods	Extinction coefficient: 0.0170 (18)
Secondary atom site location: difference Fourier map	

### Special details

**Table:**

Geometry. All e.s.d.'s (except the e.s.d. in the dihedral angle between two l.s. planes) are estimated using the full covariance matrix. The cell e.s.d.'s are taken into account individually in the estimation of e.s.d.'s in distances, angles and torsion angles; correlations between e.s.d.'s in cell parameters are only used when they are defined by crystal symmetry. An approximate (isotropic) treatment of cell e.s.d.'s is used for estimating e.s.d.'s involving l.s. planes.
Refinement. Refinement of *F*^2^ against ALL reflections. The weighted *R*-factor *wR* and goodness of fit *S* are based on *F*^2^, conventional *R*-factors *R* are based on *F*, with *F* set to zero for negative *F*^2^. The threshold expression of *F*^2^ > σ(*F*^2^) is used only for calculating *R*-factors(gt) *etc*. and is not relevant to the choice of reflections for refinement. *R*-factors based on *F*^2^ are statistically about twice as large as those based on *F*, and *R*-factors based on ALL data will be even larger.

### Fractional atomic coordinates and isotropic or equivalent isotropic displacement parameters (Å^2^)

**Table:**

	*x*	*y*	*z*	*U*_iso_*/*U*_eq_	
C1	0.1987 (2)	0.6943 (2)	0.02446 (17)	0.0490 (5)	
C2	0.1627 (3)	0.5987 (2)	−0.0454 (2)	0.0568 (6)	
C3	0.0175 (3)	0.5780 (3)	−0.1077 (2)	0.0683 (7)	
H3	−0.0065	0.5127	−0.1541	0.082*	
C4	−0.0902 (3)	0.6551 (3)	−0.0999 (2)	0.0705 (7)	
H4	−0.1879	0.6418	−0.1415	0.085*	
C5	−0.0563 (3)	0.7510 (3)	−0.0320 (2)	0.0679 (7)	
H5	−0.1304	0.8030	−0.0280	0.082*	
C6	0.0887 (3)	0.7707 (2)	0.03086 (19)	0.0547 (6)	
C7	0.4023 (2)	0.6546 (2)	0.17553 (18)	0.0499 (5)	
C8	0.5626 (2)	0.6726 (2)	0.22269 (17)	0.0484 (5)	
C9	0.6386 (3)	0.7563 (2)	0.1830 (2)	0.0636 (7)	
H9	0.5881	0.8063	0.1266	0.076*	
C10	0.7903 (3)	0.7648 (3)	0.2280 (3)	0.0744 (8)	
H10	0.8409	0.8210	0.2009	0.089*	
C11	0.8687 (3)	0.6923 (3)	0.3118 (2)	0.0693 (8)	
C12	0.7918 (3)	0.6113 (3)	0.3517 (2)	0.0681 (7)	
H12	0.8426	0.5620	0.4086	0.082*	
C13	0.6393 (3)	0.6016 (2)	0.3082 (2)	0.0574 (6)	
H13	0.5887	0.5471	0.3369	0.069*	
C14	1.0350 (3)	0.7024 (4)	0.3581 (3)	0.1081 (14)	
H14A	1.0606	0.7552	0.4189	0.130*	
H14B	1.0733	0.7342	0.3047	0.130*	
H14C	1.0766	0.6245	0.3799	0.130*	
O1	0.34465 (16)	0.71769 (15)	0.08263 (13)	0.0551 (5)	
O2	0.32703 (19)	0.59229 (19)	0.21108 (15)	0.0692 (6)	
Cl1	0.30058 (9)	0.50474 (7)	−0.05432 (7)	0.0849 (4)	
Cl2	0.13347 (8)	0.89253 (7)	0.11604 (6)	0.0796 (3)	

### Atomic displacement parameters (Å^2^)

**Table:**

	*U*^11^	*U*^22^	*U*^33^	*U*^12^	*U*^13^	*U*^23^
C1	0.0404 (11)	0.0544 (12)	0.0506 (12)	0.0032 (9)	0.0127 (9)	0.0059 (9)
C2	0.0570 (14)	0.0574 (14)	0.0578 (13)	0.0078 (10)	0.0210 (11)	0.0025 (10)
C3	0.0700 (16)	0.0723 (17)	0.0568 (14)	−0.0073 (13)	0.0124 (12)	−0.0048 (12)
C4	0.0492 (13)	0.093 (2)	0.0597 (15)	−0.0064 (13)	0.0040 (11)	0.0035 (14)
C5	0.0443 (12)	0.091 (2)	0.0654 (15)	0.0142 (12)	0.0139 (11)	0.0084 (14)
C6	0.0488 (12)	0.0627 (14)	0.0522 (12)	0.0091 (10)	0.0157 (10)	0.0002 (10)
C7	0.0464 (12)	0.0493 (12)	0.0528 (12)	−0.0002 (9)	0.0143 (9)	0.0007 (9)
C8	0.0424 (11)	0.0485 (12)	0.0538 (12)	−0.0010 (9)	0.0148 (9)	−0.0070 (9)
C9	0.0577 (14)	0.0586 (14)	0.0741 (16)	−0.0067 (11)	0.0206 (12)	0.0040 (12)
C10	0.0577 (15)	0.0775 (19)	0.090 (2)	−0.0224 (13)	0.0267 (14)	−0.0050 (15)
C11	0.0432 (13)	0.091 (2)	0.0695 (16)	−0.0092 (12)	0.0131 (11)	−0.0208 (14)
C12	0.0478 (13)	0.0830 (18)	0.0635 (15)	0.0035 (12)	0.0042 (11)	−0.0020 (13)
C13	0.0479 (13)	0.0621 (14)	0.0597 (14)	−0.0038 (10)	0.0139 (10)	−0.0018 (10)
C14	0.0438 (15)	0.161 (4)	0.110 (3)	−0.0203 (19)	0.0125 (16)	−0.025 (3)
O1	0.0408 (8)	0.0593 (10)	0.0611 (10)	0.0006 (6)	0.0109 (7)	0.0097 (7)
O2	0.0502 (9)	0.0845 (13)	0.0681 (11)	−0.0149 (8)	0.0125 (8)	0.0170 (9)
Cl1	0.0880 (6)	0.0766 (5)	0.0967 (6)	0.0244 (4)	0.0389 (5)	−0.0084 (4)
Cl2	0.0782 (5)	0.0754 (5)	0.0764 (5)	0.0184 (3)	0.0128 (4)	−0.0178 (3)

### Geometric parameters (Å, °)

**Table:**

C1—C6	1.377 (3)	C8—C13	1.379 (3)
C1—C2	1.377 (3)	C8—C9	1.385 (3)
C1—O1	1.383 (3)	C9—C10	1.382 (4)
C2—C3	1.386 (4)	C9—H9	0.9300
C2—Cl1	1.718 (2)	C10—C11	1.379 (4)
C3—C4	1.371 (4)	C10—H10	0.9300
C3—H3	0.9300	C11—C12	1.372 (4)
C4—C5	1.364 (4)	C11—C14	1.513 (4)
C4—H4	0.9300	C12—C13	1.389 (4)
C5—C6	1.384 (4)	C12—H12	0.9300
C5—H5	0.9300	C13—H13	0.9300
C6—Cl2	1.725 (3)	C14—H14A	0.9600
C7—O2	1.199 (3)	C14—H14B	0.9600
C7—O1	1.365 (3)	C14—H14C	0.9600
C7—C8	1.470 (3)		
			
C6—C1—C2	119.2 (2)	C9—C8—C7	122.4 (2)
C6—C1—O1	120.3 (2)	C10—C9—C8	119.4 (3)
C2—C1—O1	120.4 (2)	C10—C9—H9	120.3
C1—C2—C3	120.8 (2)	C8—C9—H9	120.3
C1—C2—Cl1	119.07 (19)	C11—C10—C9	121.8 (3)
C3—C2—Cl1	120.2 (2)	C11—C10—H10	119.1
C4—C3—C2	119.0 (3)	C9—C10—H10	119.1
C4—C3—H3	120.5	C12—C11—C10	118.2 (2)
C2—C3—H3	120.5	C12—C11—C14	121.3 (3)
C5—C4—C3	121.0 (2)	C10—C11—C14	120.5 (3)
C5—C4—H4	119.5	C11—C12—C13	121.0 (3)
C3—C4—H4	119.5	C11—C12—H12	119.5
C4—C5—C6	119.7 (2)	C13—C12—H12	119.5
C4—C5—H5	120.1	C8—C13—C12	120.2 (2)
C6—C5—H5	120.1	C8—C13—H13	119.9
C1—C6—C5	120.2 (2)	C12—C13—H13	119.9
C1—C6—Cl2	119.48 (19)	C11—C14—H14A	109.5
C5—C6—Cl2	120.26 (19)	C11—C14—H14B	109.5
O2—C7—O1	122.0 (2)	H14A—C14—H14B	109.5
O2—C7—C8	126.1 (2)	C11—C14—H14C	109.5
O1—C7—C8	111.85 (18)	H14A—C14—H14C	109.5
C13—C8—C9	119.3 (2)	H14B—C14—H14C	109.5
C13—C8—C7	118.3 (2)	C7—O1—C1	116.30 (17)
			
C6—C1—C2—C3	−0.9 (4)	O2—C7—C8—C9	−172.7 (3)
O1—C1—C2—C3	−177.0 (2)	O1—C7—C8—C9	7.8 (3)
C6—C1—C2—Cl1	179.23 (18)	C13—C8—C9—C10	1.7 (4)
O1—C1—C2—Cl1	3.2 (3)	C7—C8—C9—C10	−177.0 (2)
C1—C2—C3—C4	0.8 (4)	C8—C9—C10—C11	−0.2 (5)
Cl1—C2—C3—C4	−179.3 (2)	C9—C10—C11—C12	−1.0 (5)
C2—C3—C4—C5	−0.1 (4)	C9—C10—C11—C14	179.0 (3)
C3—C4—C5—C6	−0.6 (4)	C10—C11—C12—C13	0.5 (4)
C2—C1—C6—C5	0.2 (4)	C14—C11—C12—C13	−179.4 (3)
O1—C1—C6—C5	176.3 (2)	C9—C8—C13—C12	−2.2 (4)
C2—C1—C6—Cl2	−178.59 (18)	C7—C8—C13—C12	176.6 (2)
O1—C1—C6—Cl2	−2.5 (3)	C11—C12—C13—C8	1.0 (4)
C4—C5—C6—C1	0.5 (4)	O2—C7—O1—C1	−8.0 (3)
C4—C5—C6—Cl2	179.3 (2)	C8—C7—O1—C1	171.58 (18)
O2—C7—C8—C13	8.5 (4)	C6—C1—O1—C7	100.0 (3)
O1—C7—C8—C13	−171.0 (2)	C2—C1—O1—C7	−84.0 (3)
